# The Saccadic and Neurological Deficits in Type 3 Gaucher Disease

**DOI:** 10.1371/journal.pone.0022410

**Published:** 2011-07-20

**Authors:** William Benko, Markus Ries, Edythe A. Wiggs, Roscoe O. Brady, Raphael Schiffmann, Edmond J. FitzGibbon

**Affiliations:** 1 Developmental and Metabolic Neurology Branch, National Institute of Neurological Disorders and Stroke, National Institutes of Health, Bethesda, Maryland, United States of America; 2 University Children's Hospital, Pediatric Neurology, Heidelberg, Germany; 3 Institute of Metabolic Disease, Baylor Research Institute, Dallas, Texas, United States of America; 4 National Eye Institute, National Institutes of Health, Bethesda, Maryland, United States of America; Charité Universitaetsmedizin Berlin, Germany

## Abstract

Our objective was to characterize the saccadic eye movements in patients with type 3 Gaucher disease (chronic neuronopathic) in relationship to neurological and neurophysiological abnormalities. For approximately 4 years, we prospectively followed a cohort of 15 patients with Gaucher type 3, ages 8–28 years, by measuring saccadic eye movements using the scleral search coil method. We found that patients with type 3 Gaucher disease had a significantly higher regression slope of duration vs amplitude and peak duration vs amplitude compared to healthy controls for both horizontal and vertical saccades. Saccadic latency was significantly increased for horizontal saccades only. Downward saccades were more affected than upward saccades. Saccade abnormalities increased over time in some patients reflecting the slowly progressive nature of the disease. Phase plane plots showed individually characteristic patterns of abnormal saccade trajectories. Oculo-manual dexterity scores on the Purdue Pegboard test were low in virtually all patients, even in those with normal cognitive function. Vertical saccade peak duration vs amplitude slope significantly correlated with IQ and with the performance on the Purdue Pegboard but not with the brainstem and somatosensory evoked potentials. We conclude that, in patients with Gaucher disease type 3, saccadic eye movements and oculo-manual dexterity are representative neurological functions for longitudinal studies and can probably be used as endpoints for therapeutic clinical trials.

**Trial Registration:**

ClinicalTrials.gov NCT00001289

## Introduction

Gaucher disease is an autosomal recessive disorder that results from the deficiency of the lysosomal enzyme glucocerebrosidase and the accumulation of glucosylceramide in macrophages systemically in most patients [1]. The chronic neuronopathic form of Gaucher disease (type 3 Gaucher disease, GD3), is a neurological disorder that has a very variable clinical expression. It is associated with an accumulation of glucosylceramide in perivascular macrophages and in brain glial cells and in neurons [2], [3]. Recent studies in mice models for neuronopathic Gaucher disease confirmed that GD3 is primarily a neuronal disorder with an important additional role of astrocytes, while lipid laden macrophages (Gaucher cells) play a minor modulator role [4].

The hallmark clinical abnormality of patients with neuronopathic Gaucher disease consists of markedly slow horizontal saccades [3], [5]. The term saccade initiation failure or oculomotor apraxia has been used for this neurological deficit [6]. However, we and others have observed for some time that vertical saccades are slow as well but to a lesser extent and this deficit lags in time [6], [7], [8].

There is currently no effective specific treatment for the neurological aspects of Gaucher disease. High dose enzyme replacement therapy has an effect on the visceral disease, but unfortunately it has no effect on the brain [9]. For the development of future therapies it is important to longitudinally document the natural history as quantitatively as possible. We recently reported the progression of saccadic and other neurological and neuropsychological abnormalities over two years as part of a treatment trial [7]. The course of the neurological disease progression in that study was slow, therefore, it is of particular interest to further quantitate and better characterize the neurological course of GD3 over a longer period of time. Vertical saccadic function was chosen as a primary outcome measure because vertical saccades were abnormal in all our subjects but were much less affected than horizontal saccades. In some of our subjects horizontal saccades were so abnormal as to be unrecordable. Thus vertical saccade performance was felt to be a more likely parametric measure than horizontal saccades which had a floor effect. In addition to the characterization of the eye movements we investigated how the vertical saccade abnormality correlates with systemic and neurological parameters in GD3. The determination of the eye-hand coordination and oculo-manual dexterity especially was a special function of interest, because of its role in everyday life activities. Saccadic eye movements are an important component of coordination [10]. Abnormal eye-hand coordination may cause impairment of daily activities and is disturbed in a variety of other neurological conditions, such as cerebral palsy, occulomotor apraxia, Parkinson's disease, and aging [10], [11], [12], [13], [14]. Patients with GD3 were also found to demonstrate abnormal cortical inhibition compared to controls as determined by somatosensory evoked potentials [15]. Progressive abnormalities in brainstem auditory evoked responses in patients with Gaucher disease were described [16]. These findings correlated with an absence of neurons in the cochlear nuclei and a hypoplastic superior olivary complex [17]. Long-term longitudinal data of both markers have not been available yet and therefore are of particular interest.

In order to fully characterize the saccadic abnormalities in Gaucher disease, we prospectively studied the saccadic characteristics in 15 patients with GD3, with an emphasis on vertical saccades, using a reliable recording method for eye movements. To better understand the progression of the disease and the relevance of the saccadic abnormality in GD3, we also evaluated its relationship to other important brain functions and pathways.

## Materials and Methods

The protocol for this trial and supporting CONSORT checklist are available as supporting information; see Checklist S1 and Protocol S1.

### Patients

#### Ethics Statement

**Figure 1:**
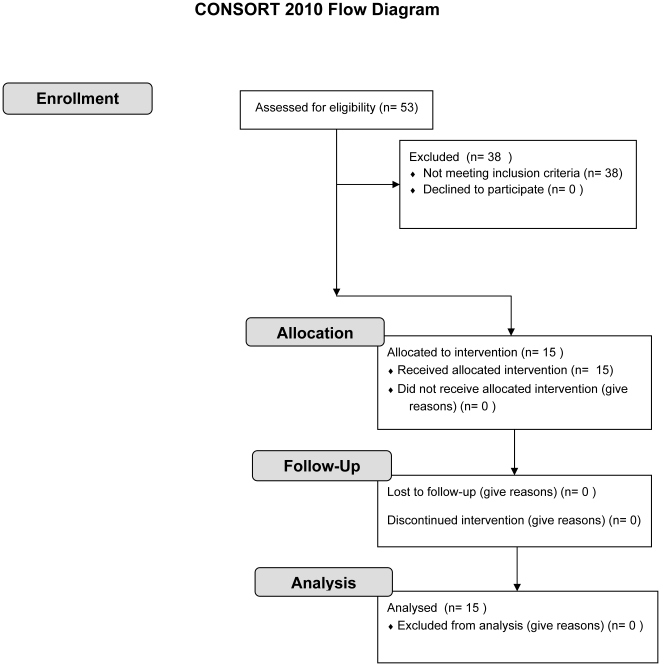
CONSORT diagram for this study.

All patients or their legal guardians gave their written informed consents. This paper consists of a description of 15 patients with Type 3 Gaucher disease (Figure 1) who were prospectively followed at the Clinical Center of the National Institutes of Health on an approved clinical research protocol of the National Institute of Neurological Disorders and Stroke, NIH (ClinicalTrials.gov identifier: NCT00001289). The criteria for inclusion in this report were patients who were studied on more than one visit using the scleral search coil method for assessing saccadic eye movements (see below).

The patients were 8 males and 7 females with a mean age of 15.7±5.4 (age range 8 to 28). For eye movement studies, patients were followed for a mean of 2.3±1.0 years. A normal control group was composed of 10 subjects: 5 males and 5 females with a mean age of 16.7 years (age range 11 to 31).

### Eye Movements

**Figure 2:**
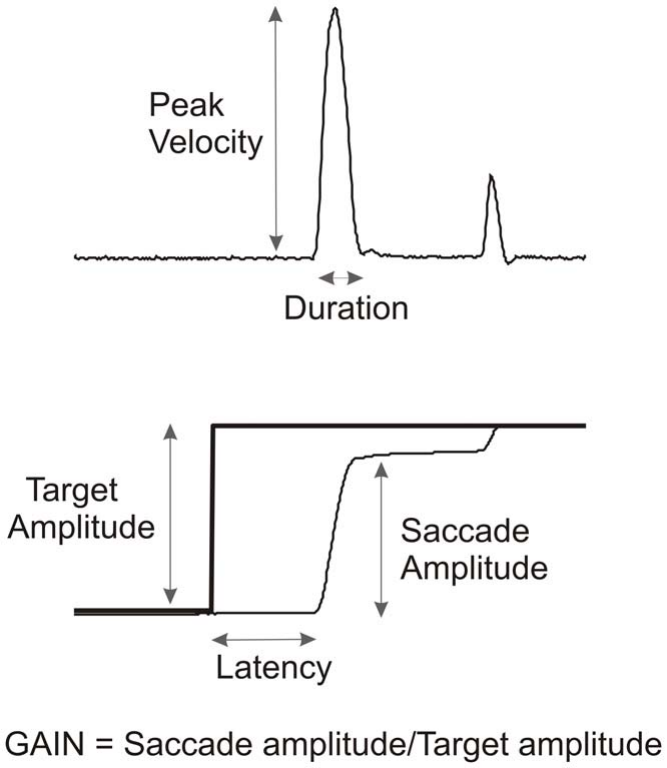
Saccadic eye movement parameters.

Patients had a general ophthalmologic assessment before assessment of saccadic eye movements, to identify visual impairment. Horizontal and vertical saccadic eye movements were recorded using a scleral search coil technique and were sampled at 1 kHz using Realtime (EXperimentation) (REX) [18]. The subjects sat with their head in a chin cup and their forehead against a headrest and faced a screen 1 meter away on which a red laser spot was back projected (the spot subtended about 0.5 deg). A mirror galvanometer moved the target spot in a pseudorandom sequence from -15 deg to +15 deg with target jumps of 2.5, 5, 7.5, 10, 12.5, 15, 20, 25, 30 deg in each direction. The target jumped approximately every 3 seconds with about 0.5 sec variability. Subjects were instructed to follow the target with their eyes. A minimum of 100 target jumps was recorded for a sequence of vertical saccades and then a minimum of 100 target jumps for horizontal saccades. The eye position data was then filtered and differentiated to obtain eye velocity. Saccades were detected using a velocity criterion, and their characteristics (latency, amplitude, duration, and peak velocity) were determined (Figure 2). Saccade latency is defined as the time from target onset to the time of eye motion onset using velocity criteria (Figure 2). Saccade gain is defined as the amplitude of the first saccade made to a target divided by the target's distance from fixation (Figure 2). Saccade peak duration is defined as saccade amplitude divided by saccade peak velocity and was used to linearize the graph of saccade velocity vs amplitude, often referred to as the main sequence [18], [19], [20]. The graph of saccade duration vs amplitude tended to be linear and further manipulations were not necessary [18], [20]. Regression lines were fitted to the data of saccade peak duration vs amplitude and saccade duration vs amplitude as a method to summarize saccade performance for any particular recording session. Slower, more abnormal saccades yielded regression slopes that were larger for duration vs amplitude graphs and larger for peak duration vs amplitude graphs.

### Methods of evaluating systemic disease

Skeletal abnormalities were assessed using plain films of the long bones. Hematological parameters as well as pulmonary status, spine, liver and spleen volume were assessed as previously described [21].

### Methods of evaluating neurological disease

Neuropsychological assessments included Purdue Pegboard test and Wechsler scale administered as part of a larger battery of neuropsychological tests that included measures of attention and memory. Only the results of the Wechsler IQ scales and the Purdue Pegboard test are presented here. The Wechsler scales are widely used, standardized IQ tests. The Purdue Pegboard test is a validated test of manual dexterity and eye-hand coordination. It consists of a board, approximately 30.5 cm×45.5 cm, with two parallel rows of 25 holes each. Pegs are located at the top of the board in wells, one to the left and one to the right. Between the peg wells are two additional wells containing washers and collars. In the first of 4 trials, the subject removes one peg from the well on the same side as the dominant (observed writing) hand and places the peg in the first hole on the same side. A short practice is required, and then the trial is timed for 30 seconds. In the second of the 4 trials, the procedure is repeated with the nondominant hand. In the third trial the subject removes a peg from each well simultaneously and places the pegs in the holes on the respective side of the board. In the fourth and last trial, the subject takes a peg with the dominant hand, while simultaneously taking a washer with the nondominant hand and placing the peg in the top hole on the dominant hand side, slipping the washer onto the peg. With the dominant hand a collar is retrieved, placed on the washer while a second washer is retrieved with the nondominant hand and placed atop the collar. This completes one assembly. The subject is given 60 seconds to make as many 4-part assemblies as possible. Number of correctly placed pegs (for trials 1 through 3) and number of pieces correctly assembled are compared to published norms and expressed in z-scores [22], [23]. Standard EEG, brainstem auditory evoked response (BAER), somatosensory evoked potentials (SSEP) using median nerve stimulation and stretch-evoked methods, as previously described, were performed on each subject at each visit to the NIH. In the stretch-evoked SSEP, the muscle stretch was produced by tapping the subject's hand with a plastic hammer [15]. Absolute and inter-peak latency values were obtained from the BAER, and P1-N2 amplitude data were obtained from the SSEP. BAER and EEG findings were categorized into clinical severity scores from 0 to 5 (higher numbers indicating greater abnormality).

### Statistical methods

Correlation analyses using Spearman's rank correlation coefficient were conducted between the slopes of peak duration vs amplitude for vertical saccades of patients and each of the neuropsychological parameters and systemic parameters, respectively, with a two-tailed significance level of 0.05. Similarly, correlation analyses for horizontal saccade regression slope and vertical saccade regression slope were done respectively.

To control for the effect of age at saccadic testing, Spearman's partial rank correlation coefficient was used to explore correlation between any pair of BAER or SSEP and vertical or horizontal slope.

To explore the time effect on each of the neurological, non-neurological or systemic parameters except for BAER score and SSEP amplitude, multivariate analysis of variance (MANOVA) models with time as a within factor were used. Time effect on BAER score or SSEP amplitude was examined using a multivariate analysis of covariance (MANCOVA) with time as a within factor and age at saccadic testing as a covariate. The covariance structure was assumed to be first order auto regressive covariance structure while using the Kenward-Roger degrees of freedom method in both MANOVA and MANCOVA.

Analyses were implemented in SAS PROC Corr and PROC Mixed (SAS Institute Inc., Cary, North Carolina: Littell et al. 1996) with α = 0.05.

## Results

### Patient characteristics

The 15 patients were followed over 2–4 years with repeated testing. General patient characteristics are described in Table S1. Eleven of the patients were homozygotes for the common L444P mutation and together with patient 5 they presented with typical stable chronic neuronopathic clinical abnormalities with underlying severe systemic disease (Table S2). Patient 2 had progressive myoclonic encephalopathy with seizures. All patients except one were on enzyme replacement therapy (ERT) with imiglucerase for years, while patient 14 was successfully treated with hematopoietic stem cell transplantation. Ten patients temporarily received investigative substrate reduction therapy (OGT 918, miglustat) as part of a clinical trial [7].

### Saccadic eye movements abnormalities

In examining the saccades of patients we focused particularly on the relationship between peak duration (defined as peak velocity/amplitude) and amplitude using the slope which was calculated by linear regression fit of the saccades [19].

All patients in this current study had abnormal saccadic velocities with vertical saccades less affected than horizontal saccades. In 9 of 15 patients, the downward saccades were slower than upward ones. Comparing the slopes of amplitude vs duration for downward vs upward saccades for the patients, downward saccades were significantly longer in each patient (9.15±5.72 vs 5.21±2.02 with p<0.01) and more variable. Slopes of peak duration vs amplitude of downward saccades were also significantly greater than upward saccades (4.67±3.66 vs 2.55±1.04 with p<0.04) and this was noted in 13 of 15 patients.

The latency of horizontal saccades was longer than vertical saccades in patients and significantly slower than the vertical saccades of normal controls (Table 1). However, the latency of vertical saccades of patients was only slightly longer than normal. The gain of horizontal saccades was lower than vertical in patients and gains for both horizontal and vertical saccades were significantly smaller than in normal controls (Table 1). Studying the slopes of regression of saccade duration vs amplitude, horizontal saccades of patients were most affected, followed by downward saccades and least affected were upward saccades. All slopes of saccade duration vs amplitude fits were larger than for healthy controls (Table 1). Comparing the slopes of saccade peak duration vs amplitude, the same pattern emerged (Table 1).

**Table 1:** Saccade Characteristics. Comparison of saccade parameters of patients and normal controls using data from their last visit.

Saccade Latency	Gaucher	Normal	T test
Right	0.34±0.14	0.20±0.03	0.004
Left	0.32±0.10	0.20±0.02	0.002
Up	0.25±0.09	0.21±0.03	0.24
Down	0.25±0.06	0.23±0.06	0.52

Saccade Gain	Gaucher	Normal	T test
Right	0.54±0.17	0.89±0.07	2.5×10^−6^
Left	0.57±0.22	0.89±0.06	0.0001
Up	0.82±0.19	0.88±0.10	0.36
Down	0.73±0.19	0.90±0.06	0.015

Slope of Duration vs Amplitude	Gaucher	Normal	T test
Right	13.87±6.15	2.71±0.43	4.9×10^−6^
Left	14.20±6.64	2.66±0.52	9.1×10^−6^
Up	5.21±2.02	3.16±0.93	0.006
Down	9.15±5.72	3.41±0.97	0.005

Slope of Peak Duration vs Amplitude	Gaucher	Normal	T test
Right	7.69±2.45	1.62±0.29	3.1×10^−8^
Left	9.39±4.63	1.67±0.45	1.6×10^−5^
Up	2.55±1.04	1.75±0.62	0.04
Down	4.67±3.65	1.79±0.50	0.02

**Figure 3:**
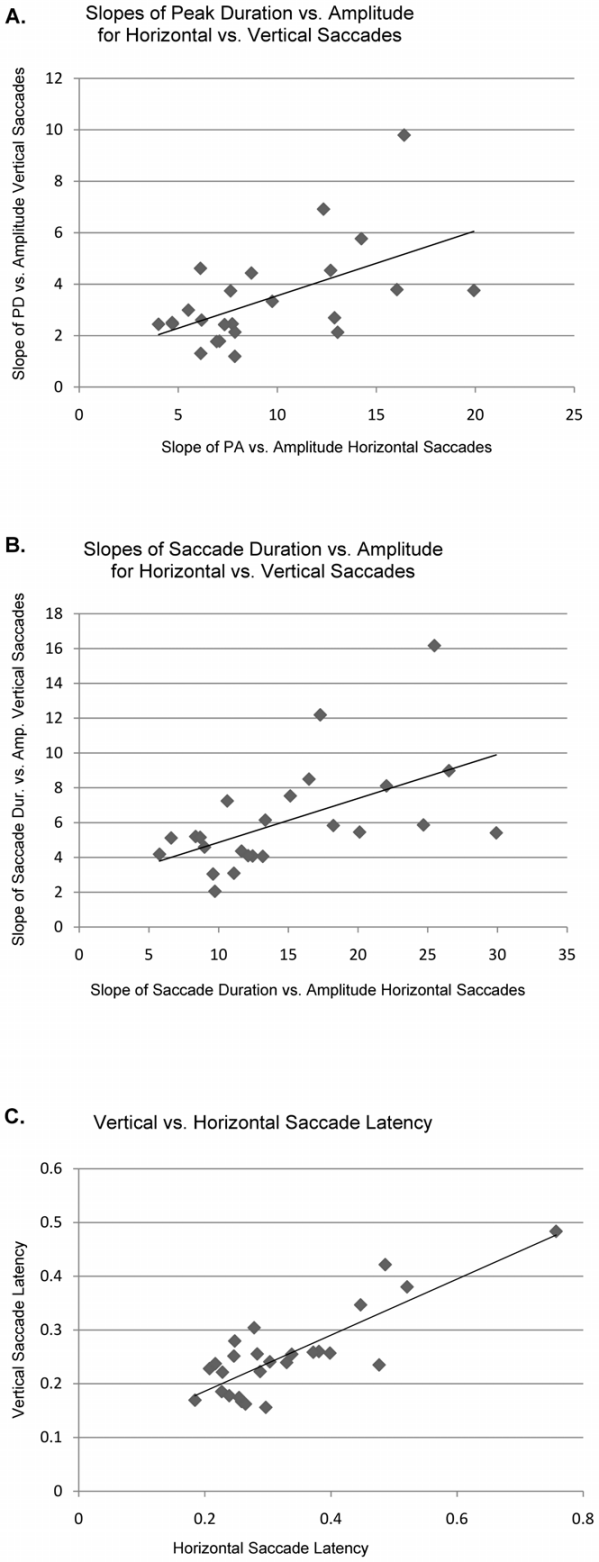
A. Slope of peak duration vs amplitude for vertical vs horizontal saccades. The line is a least squares regression of the data points and has an R^2^ value of 0.3. This graph suggests there is a tendency for vertical and horizontal saccade performance to track together and the p value for that tendency is <0.005. **B.** Graph of regression slopes of saccade duration vs amplitude for vertical vs horizontal saccades. Again the least squares regression fit of the data points is shown and has an R^2^ value of 0.31. The test for relationship has a p<0.005. **C.** Graph of vertical vs horizontal latencies (data from the last visit). The regression line has an R^2^ of 0.68 and the test for relationship has p<0.00001.

**Figure 4:**
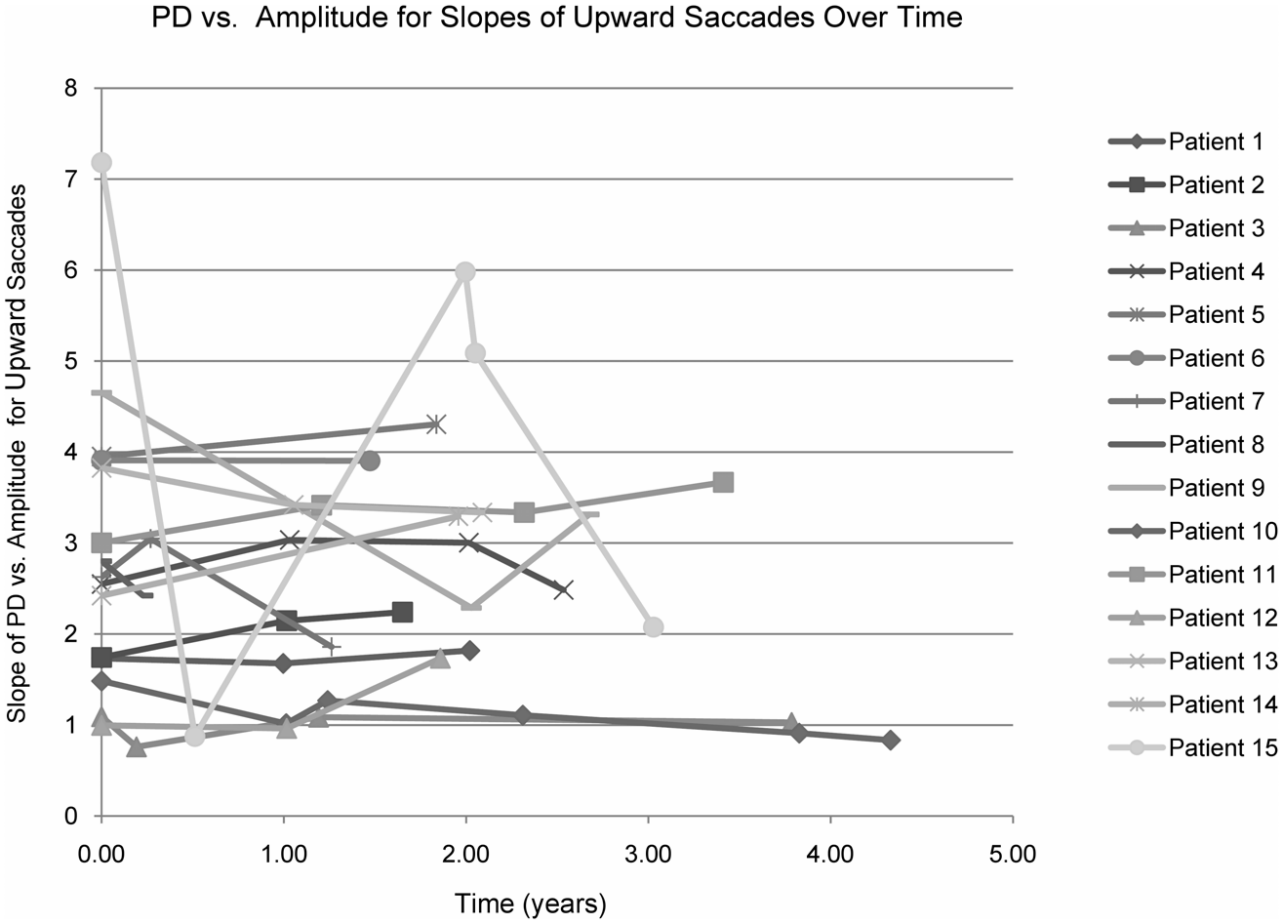
Slope of peak duration vs amplitude for upward saccades over time. Each point represents the results for a specific visit time.

We asked whether patients with more severely affected horizontal saccades would have more severely affected vertical saccade parameters. Graphing the slopes of horizontal vs vertical saccades for duration vs amplitude and peak-duration vs amplitude we found correlation coefficients of 0.31 in both cases (p<0.005, Figure 3A, 3B). The regression of horizontal vs vertical saccade latency had a correlation coefficient of 0.68 (p<0.005, Figure 3C). This suggests that the two systems do tend to track together, both worsening with disease progression. However, the correlation is imperfect, in part because there is a floor effect when the horizontal saccades are very poor and cannot become any worse. For several patients with the longest follow up, we viewed saccadic parameters across time and were unable to demonstrate a consistent decline in follow up visits by looking at the slopes of peak duration vs amplitude for each patient's upward saccades over time (Figure 4). Upward saccades were chosen since these were the least abnormal. Graphing downward and horizontal saccade slopes over time did not demonstrate any consistent pattern of decline (see also below).

**Figure 5:**
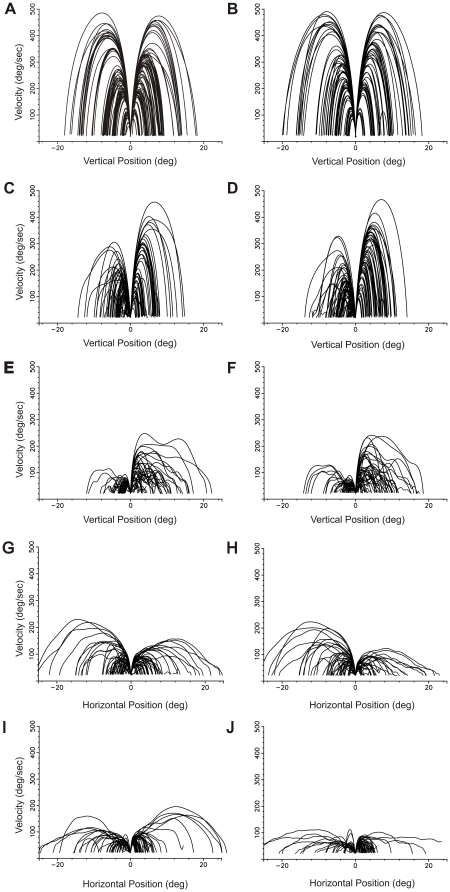
Phase plane plots for saccadic velocity vs position. These are phase plane plots of saccades made by 5 subjects on a specific date to differing target amplitudes. The origin of each saccade has been moved to zero and the velocity at each position along the saccade trajectory is shown. Since larger amplitude saccades typically generate faster velocities, this generates a family of curves where the saccade amplitude can be seen at the endpoint position. Here rightward curves are upward (A–F) or rightward (G–J) saccades and leftward saccades are downward (A–F) or leftward (G–J) saccades. A and B are plots of vertical saccades for 2 different normal controls. Horizontal saccades for normal controls were also similar to these plots and to each of the other normal controls. C and D show vertical saccades for Patient 3 at 2 different time points: D is 4 years later than C. E and F are similar vertical saccade plots for Patient 5, who had more difficulty making saccades. F is 4 years later than E. Note that for both patients downward saccades are slower than upward saccades. The plots in E and F also demonstrate the tendency for saccades to slow down and speed up during the course of the saccade. Although phase plane plots of normal controls' saccades looked similar to each other, each patient tended to have uniquely appearing phase plots that were reproducible over time as seen in these 2 patients. G through J are plots of horizontal saccadic velocity vs position. G and H are horizontal saccade phase plane plots for Patient 3, whose vertical saccades are plotted in C and D. Note that the horizontal saccades are slower here and tend to have more periods of slowing than for the same patient's vertical saccades. Again there is a 4-year time period between G and H. I and J are horizontal saccades for Patient 11 and there is a 4-year time period between J and I. These horizontal saccades are slower than in Patient 3 in A and B. In the later plot of both patients there is a tendency for the horizontal saccades to be slower over the 4-year period of observation.

Patients tended to have individually characteristic patterns of saccades when viewing phase planes of position vs velocity, and these unique patterns remained over time. In contrast, phase planes of the saccades of normal controls tended to resemble each other (Figure 5).

### Other Systemic and Neurological Characteristics

Hematological parameters and organ volumes at each testing visit as well as whether or not the patient was on OGT 918 (miglustat) are described in Table S2. In a recent randomized controlled trial, miglustat had no effect on saccadic velocity[7]. Platelet counts and hemoglobin levels were normal or near normal in all patients. Neuropsychological testing and neurophysiological testing at each visit (when available) are described in Tables S3 and S4 respectively. IQ ranged from well above average to severe mental impairment (full scale IQ ranges from 124 to 40). Remarkably, the performance on the Purdue Pegboard test was generally abnormal even in patients with normal cognitive function (Table S3). Only four patients had completely normal BAER (Table S4). Patients 2 and 4 had elevated SSEP amplitude both by median nerve stimulation and by stretch reflex (hammer) as seen in patients with progressive myoclonic encephalopathy of Gaucher disease. Four patients had partial complex seizures with or without secondary generalization that was not associated with elevated SSEP amplitude, suggesting a focal onset that is not part of a progressive epileptic encephalopathy.

### Change Over Time and Correlations

To explore the time effect on each of the neurological and non-neurological (systemic) parameters we found that only liver volume had a significant time effect (p<0.01). In order to assess whether the severity of the saccadic abnormalities reflects the overall impairment in GD3, we investigated possible correlations between the severity of the saccadic eye movement deficit and the systemic, neurophysiologic and neuropsychological parameters (Tables 1 and S3-S4) using Spearman's rank correlation coefficient. When looking at the initial record of each patient, significant correlation was found between Verbal IQ (p<0.02), Performance IQ (p<0.04), Full-Scale IQ (p<0.02) and the downward saccade slope. Significant correlation was found between the vertical saccade peak duration slope and the performance of the patients on the Purdue Pegboard when the patient used both hands (trial 3) (p = 0.01). There was no significant correlation between the BAER and SSEP and the saccadic eye movement parameter except for BAER on the right side (p = 0.04).

## Discussion

In this paper we describe the various characteristics and the natural history during a 4-year follow up of the saccadic eye movement abnormalities in patients with GD3 and their relationship to other aspects of neurological function.

Horizontal saccadic abnormalities are the hallmark of GD3 and all our patients also demonstrated both horizontal and vertical saccadic abnormalities. Optokinetic nystagmus and vestibular nystagmus quick phases show similar slowness as do saccades [5], [6], [8].

The pathophysiology underlying the saccade abnormalities in neuronopathic Gaucher disease is unclear. Several brain areas are involved with saccade generation including the cerebellum, basal ganglia and brainstem. The brainstem areas attributed to saccadic control include the parapontine reticular formation (PPRF) for horizontal saccades and the midbrain rostral interstitial nucleus of the medial longitudinal fasciculus (riMLF) for vertical saccades [10]. Our study showed that although vertical saccade lagged horizontal saccade abnormalities, both tended to deteriorate together. We might expect to see less disease involvement in the riMLF than in the PPRF on autopsy studies. However, in neuronopathic Gaucher autopsy material the brainstem shows widespread gliosis and neuronal loss making it hard to distinguish discrete nuclei in the advanced disease state [2]. It is interesting that downward saccades were often more affected than upward saccades and this has also been noted in parkinsonian syndromes such as progressive supranuclear palsy and other neuro-degenerative diseases with vertical saccade abnormalities [24]. This likely represents differential disease effects on the vertical saccade generation pathways.

The phase plane plots of Figures 3 and 4 reveal some interesting aspects of saccade performance in these patients. In comparison to normal controls who produce a smooth family of curves for different saccade amplitudes, each patient has a somewhat idiosyncratic and characteristic family of velocity trajectories, often with slowing and speeding up of the saccade during its flight, which appears as wiggles in the curve. These velocity variations are more often present for larger amplitude saccades and for patients with more severely affected saccades. This may reflect problems with turning off cells in the brainstem saccade omnipause region, which might be interfering with the saccade progress [10]. Another aspect that can be seen in these patients' phase plane plots is that there were fewer large amplitude saccades produced (reflecting decreased gain) and the peak velocity during any given saccade was smaller. Unlike normal controls whose phase planes look similar to each other, each patient's saccade performance created a fairly unique velocity vs position phase plane and retained its individuality over time. This might represent unique patterns of differential degeneration in individuals affecting their saccade generation. Lastly, there was some suggestion of saccade performance deterioration over the 4 years time course in the two examples of Figure 3, suggesting disease progression.

The significant correlation between the most prominent measurable saccadic abnormalities and other neurological parameters (IQ and brainstem auditory function) suggests a correlation between cerebral disease state (as reflected in IQ) and brainstem pathology (as reflected in saccade velocity). Therefore, measurement of saccadic eye movements may be an adequate representation of the neurological deficit in GD3 and may be a useful biomarker as part of a neuropsychological and neurophysiological testing battery which should also include IQ measurements in future therapeutic clinical trials. Although not a sufficient criterion, the presence of a significant correlation between the biomarker and the ‘true’ clinical endpoint is necessary to validate surrogate endpoints in clinical trials [25], [26]. The absence of a comprehensive correlation between all the saccadic eye movement parameters and the systemic, neuropsychological and neurophysiological characteristics may be due to the small number of subjects along with the variability of the test results, but it also indicates the variability of clinical deficits and disease phenotype among our patients. For example, patients 3 and 12 had normal to high cognitive function, yet had saccadic deficits that were not unlike those of patients with cognitive impairment.

Saccadic eye movements have been used as primary clinical outcome measures in two recent clinical trials [7], [27]. They are useful because of their reproducibility, easy quantification and because symptomatic patients with Niemann-Pick disease type C or neuronopathic Gaucher disease have slow saccades, although patients with Niemann-Pick disease type C mainly have vertical saccadic initiation failure [27]. Deficits in saccadic eye movements limit activities such as using a computer, driving, crossing the street or going up or down stairs. The absence of significant change overall in saccadic velocity over the follow up period suggests that in this form of Gaucher disease the pathological process is very slow. However, there was a clear decline over time in some patients that was similar to the decline observed in auditory brainstem responses [16].

Similar to the saccades, the absence of statistically significant change within the neurophysiological and neuropsychological endpoints over the observed time was probably caused by a combination of very slow change, relatively short follow up period and small sample size.

In this study we also validated the stretch reflex method as a useful approach for recording SSEP in a more physiological manner [15]. This method is particularly useful for small children since it does not involve the unpleasant sensation related to electrical median nerve stimulation[15].

This study has several limitations. All patients were referred to a research institution and may not reflect the general population of patients with GD3. In addition, although we describe the natural history of the encephalopathy associated with GD3, the patients in this study are not, strictly spoken, ‘untreated’. However, neither ERT nor miglustat have demonstrated any clinical benefit on the neurological outcome in GD3.

The net effect of ERT cannot be assessed since all patients are put on ERT because it has a marked effect on the non-neurological aspects of neuronopathic Gaucher disease [21]. Although theoretically possible, it is unlikely that ERT has some salutary effect on saccades, because it is not believed to cross the blood-brain barrier. In addition, it is possible that the cells involved in saccade generation are not simply dysfunctional but rather have been killed by the Gaucher disease process and in that case the best we would expect is that treatment would prevent further progression and the saccade parameters would remain stable over time. We could not demonstrate that the use of miglustat had an influence on the progression of the saccadic eye movement abnormalities, in part because there was not much change in saccade parameters over the observed period [7].

In addition to slow saccadic eye movements, we found that virtually all patients with neuronopathic Gaucher disease, even those with normal cognition and neurological examination, have a deficit in eye-hand coordination as documented in the Purdue Pegboard test. In view of the lack of uniform correlation with vertical saccadic eye movement parameters, this manual dexterity deficit is not caused by the supranuclear gaze palsy but likely by a cortical/subcortical dysfunction. This finding suggests involvement of other centers such as the superior colliculus that is implicated in saccade selection and gaze anchoring during natural reaching movements critical for adequate eye-hand coordination [28], [29]. Therefore, the almost uniform Purdue Pegboard abnormality constitutes a novel potential clinical outcome measure in clinical trials of patients with GD3.

In summary, it is possible to study the longitudinal course of a complex neurometabolic disorder such as Gaucher disease type 3 with objective neurological, neurophysiological and neuropsychological endpoints. Our findings indicated that the saccadic and eye-hand coordination abnormalities are adequate representations of the overall neurological impairment in GD3 and may be useful as clinical outcome measures in future clinical trials.

## Supporting Information

Table S1 **Patient general characteristics.** Bone disease classifications: Mild, radiologic abnormalities and/or occasional mild pain; Moderate, fractures (including avascular necrosis) and/or chronic pain; Severe, surgery and/or disability due to pain Abbreviations: NA, not available; ILD, interstitial lung disease; PH, pulmonary hypertension; PF, pulmonary fibrosis; ME, myoclonic encephalopathy, FTT, failure to thrive; BMT, bone marrow transplant; ERT, enzyme replacement therapy, every 2 weeks dose.
(DOCX)

Table S2 **Systemic disease characteristics.** When there is no entry, no data are available for that time point.
(DOCX)

Table S3 **Neuropsychological testing.**
(DOCX)

Table S4 **Neurophysiological characteristics.** Legend for BAER score: 0-Normal absolute & normal interpeak interval 1-1- Late absolute, normal interpeak interval 2- Late absolute, Prolonged interpeak interval 3- No peaks after wave 3 4- No peaks after waves 1 or 2 5- No peaks Legend for EEG score: 0- normal 1- Diffuse background slowing without low voltage 2- Diffuse irregular background slowing with low voltage 3- Sharps and/or spike/slow wave complex(es) (diffuse and or lateralized) 4- 1+3 5- 2+3 PCS: partial complex seizures.
(DOCX)

Checklist S1 CONSORT 2010 checklist of information to include when reporting a randomised trial.
(DOC)

Protocol S1 Protocol Title: Clinical and Biochemical Effects of Macrophage-Targeted Glucocerebrosidase on Neurological Involvement in Neuronopathic Gaucher Disease'.
(DOC)

## References

[1] Brady RO, Kanfer JN, Bradley RM, Shapiro D (1966). Demonstration of a deficiency of glucocerebroside-cleaving enzyme in Gaucher's disease.. J Clin Invest.

[2] Wong K, Sidransky E, Verma A, Mixon T, Sandberg GD (2004). Neuropathology provides clues to the pathophysiology of Gaucher disease.. Mol Genet Metab.

[3] Schiffmann R, Vellodi A, Futerman AH, Zimran A (2007). Neuronopathic Gaucher Disease.. Gaucher disease.

[4] Enquist IB, Lo Bianco C, Ooka A, Nilsson E, Mansson JE (2007). Murine models of acute neuronopathic Gaucher disease.. Proc Natl Acad Sci U S A.

[5] Garbutt S, Harwood MR, Harris CM (2001). Comparison of the main sequence of reflexive saccades and the quick phases of optokinetic nystagmus.. Br J Ophthalmol.

[6] Harris CM, Taylor DS, Vellodi A (1999). Ocular motor abnormalities in Gaucher disease.. Neuropediatrics.

[7] Schiffmann R, Fitzgibbon EJ, Harris C, DeVile C, Davies EH (2008). Randomized, controlled trial of miglustat in Gaucher's disease type 3.. Ann Neurol.

[8] Garbutt S, Harris CM (2000). Abnormal vertical optokinetic nystagmus in infants and children.. Br J Ophthalmol.

[9] Vellodi A, Tylki-Szymanska A, Davies EH, Kolodny E, Bembi B (2009). Management of neuronopathic Gaucher disease: revised recommendations.. J Inherit Metab Dis.

[10] Leigh RJ, Kennard C (2004). Using saccades as a research tool in the clinical neurosciences.. Brain.

[11] Saavedra S, Joshi A, Woollacott M, van Donkelaar P (2009). Eye hand coordination in children with cerebral palsy.. Exp Brain Res.

[12] Myung JY, Blumstein SE, Yee E, Sedivy JC, Thompson-Schill SL (2010). Impaired access to manipulation features in Apraxia: evidence from eyetracking and semantic judgment tasks.. Brain Lang.

[13] Sacrey LA, Travis SG, Whishaw IQ (2011). Drug treatment and familiar music aids an attention shift from vision to somatosensation in Parkinson's disease on the reach-to-eat task.. Behav Brain Res.

[14] Rand MK, Stelmach GE (2011). Effects of hand termination and accuracy requirements on eye-hand coordination in older adults.. Behav Brain Res.

[15] Garvey MA, Toro C, Goldstein S, Altarescu G, Wiggs EA (2001). Somatosensory evoked potentials as a marker of disease burden in type 3 Gaucher disease.. Neurology.

[16] Campbell PE, Harris CM, Vellodi A (2004). Deterioration of the auditory brainstem response in children with type 3 Gaucher disease.. Neurology.

[17] Lacey DJ, Terplan K (1984). Correlating auditory evoked and brainstem histologic abnormalities in infantile Gaucher's disease.. Neurology.

[18] Inchingolo P, Spanio M (1985). On the identification and analysis of saccadic eye movements--a quantitative study of the processing procedures.. IEEE Trans Biomed Eng.

[19] Hays AV, Richmond BJ, Optican LM (1982). A UNIX-based multiple process system for real-time data acquisition and control.. WESCON Conference Proceedings.

[20] Inchingolo P, Spanio M, Bianchi M, O'Regan JK, Levy-Shoen A (1987). The characteristics peak velocity - mean velocity of saccadic eye movements in man.. Eye movements: from physiology to cognition.

[21] Altarescu G, Hill S, Wiggs E, Jeffries N, Kreps C (2001). The efficacy of enzyme replacement therapy in patients with chronic neuronopathic Gaucher's disease.. J Pediatr.

[22] Yeudall LT, Fromm D, Reddon JR, Stefanyk WO (1986). Normative data streatified by age and sex for 12 neuropsychological tests Journal of Clinical Psychology.

[23] Gardner RA, Broman M (1979). The Purdue Pegbord: Normative data on 1334 school children.. Journal of Clinical Child Psychology.

[24] Bhidayasiri R, Riley DE, Somers JT, Lerner AJ, Buttner-Ennever JA (2001). Pathophysiology of slow vertical saccades in progressive supranuclear palsy.. Neurology.

[25] Fleming TR, DeMets DL (1996). Surrogate end points in clinical trials: are we being misled?. Ann Intern Med.

[26] Prentice RL (1989). Surrogate endpoints in clinical trials: definition and operational criteria.. Stat Med.

[27] Patterson MC, Vecchio D, Prady H, Abel L, Wraith JE (2007). Miglustat for treatment of Niemann-Pick C disease: a randomised controlled study.. Lancet Neurol.

[28] Reyes-Puerta V, Philipp R, Lindner W, Hoffmann KP (2010). Role of the rostral superior colliculus in gaze anchoring during reach movements.. J Neurophysiol.

[29] Nummela SU, Krauzlis RJ (2010). Inactivation of primate superior colliculus biases target choice for smooth pursuit, saccades, and button press responses.. J Neurophysiol.

