# Supplementary material for: The Saccadic and Neurological Deficits in Type 3 Gaucher Disease
Source: PLoS One. 2011 Jul 20;6(7):e22410. doi: 10.1371/journal.pone.0022410 (PMC3140522; doi:10.1371/journal.pone.0022410)
Supplement: Table S1 — Patient general characteristics. Bone disease classifications: Mild, radiologic abnormalities and/or occasional mild pain; Moderate, fractures (including avascular necrosis) and/or chronic pain; Severe, surgery and/or disability due to pain Abbreviations: NA, not available; ILD, interstitial lung disease; PH, pulmonary hypertension; PF, pulmonary fibrosis; ME, myoclonic encephalopathy, FTT, failure to thrive; BMT, bone marrow transplant; ERT, enzyme replacement therapy, every 2 weeks dose. (DOCX) [file pone.0022410.s001.docx]

Table S1. Patient general characteristics

| Patient no. | Genotype | Sex | Ethnic background | Age at diagnosis (months) | Age at last follow-up (years) | Bone disease | Lung disease | Other systems | Splenectomy (years) | Treatment | Enzyme therapy (dose and duration) |
| --- | --- | --- | --- | --- | --- | --- | --- | --- | --- | --- | --- |
| 1 | L444P/L444P | M | Hispanic | 24 | 8 | Mild | ILD | None | No | ERT | 60 IU/kg since age 27 mo |
| 2 | P122S/P122S | M | Native American | 24 | 15 | Mild | ILD | None | No | ERT | 60 IU/kgSince age 2 |
| 3 | L444P/L444P | F | White Caucasian | 24 | 17 | None | ILD | None | No | ERT | 60 IU/kg since age 3 |
| 4 | G377S/R463Q | M | Spanish | 18 | 9 | None | None | None | No | ERT | 60 IU/kg since age 2 |
| 5 | L444P/L444P | F | African American | 4 | 12 | Mild | ILD | None | No | ERT | 60 IU/kg since age 6mo |
| 6 | L444P/L444P | M | Hispanic | 12 | 9 | None | ILD | None | No | ERT | 60 IU/kg since age 14 months |
| 7 | L444P/L444P | M | African American | 12 | 12 | Moderate | None | None | 8 | ERT | 120 IU/kg since age 15 months |
| 8 | R463C/Rec NciI+Rec 7 | F | White Caucasian | 48 | 28 | Moderate | None | None | 12 | ERT | 60 IU/kg since age 17 |
| 9 | L444P/L444P | M | White Caucasian | 15 | 12 | Mild | ILD | None | No | ERT | 40 IU/kg at age 30 months, increased to 60IU/kg |
| 10 | L444P/L444P | F | White Caucasian | 6 | 10 | None | None | None | No | ERT | 120 IU/kg since age 2 |
| 11 | L444P/L444P | M | White Caucasian | 30 | 16 | None | None | None | No | ERT | 60 IU/kg since age 3 |
| 12 | L444P/L444P | F | White Caucasian | 15 | 21 | Mild-moderate | ILD, clubbing | None | No | ERT | 60 IU/kg since age 8 |
| 13 | L444P/L444P | F | Hispanic | 24 | 18 | Mild-moderate | None | None | 2.5 | ERT | 60 IU/kg since age 3 |
| 14 | L444P/L444P | F | Hispanic | 15 | 19 | Severe | ILD | Renal involvement due to GVH | 3 | BMT | None |
| 15 | L444P/L444P | M | White Caucasian | 16 | 21 | Moderate | None | None | 2 | BMT, then ERT | 60 IU/kg since age 9 |

Bone disease classifications: Mild, radiologic abnormalities and/or occasional mild pain; Moderate, fractures (including avascular necrosis) and/or chronic pain; Severe, surgery and/or disability due to pain

Abbreviations: NA, not available; ILD, interstitial lung disease; PH, pulmonary hypertension; PF, pulmonary fibrosis; ME, myoclonic encephalopathy, FTT, failure to thrive; BMT, bone marrow transplant; ERT, enzyme replacement therapy, every 2 weeks dose.
