# Supplementary material for: The Saccadic and Neurological Deficits in Type 3 Gaucher Disease
Source: PLoS One. 2011 Jul 20;6(7):e22410. doi: 10.1371/journal.pone.0022410 (PMC3140522; doi:10.1371/journal.pone.0022410)
Supplement: Table S2 — Systemic disease characteristics. When there is no entry, no data are available for that time point. (DOCX) [file pone.0022410.s002.docx]

**Table S2. Systemic disease characteristics**

| Patient Number (year) | Spleen Volume (mL) | Liver Volume (mL) | Hemoglobin (g/dL) | Platelets (x 100,000) | On OGT918 (0= no, 1= yes) | Dates on OGT918 |
| --- | --- | --- | --- | --- | --- | --- |
|  |  |  |  |  |  |  |
| 1 (2004) | 113 | 569 | 10.9 | 172 | 0 |  |
| 1 (2005) | 104 | 632 | 11.9 | 235 | 1 |  |
| 1 (2006a) | 89 | 632 | 11.9 | 206 | 1 | 06/2004-10/2006 |
|  |  |  |  |  |  |  |
| 2 (2004) | 309 | 1139 | 13.4 | 132 | 0 | 07/2004-10/2005 |
| 2 (2005) | 365 | 989 | 13.5 | 140 | 1 |  |
| 2 (2006) | 370 | 990 | 13.2 | 168 | 1 |  |
|  |  |  |  |  |  |  |
| 3 (2002) |  |  | 14.1 | 211 | 0 |  |
| 3 (2003) |  |  | 14 | 228 | 1 | 01/2003-01/2004 |
| 3 (2004) |  |  | 14.3 | 211 | 0 |  |
| 3 (2006) |  |  | 13.9 | 212 | 1 |  |
|  |  |  |  |  |  |  |
| 4 (2004) | 253 | 925 | 11.4 | 246 | 0 | 05/2004-8/2006 |
| 4 (2005) |  |  | 11.9 | 242 | 1 |  |
| 4 (2006a) | 212 | 903 | 11.9 | 247 | 1 |  |
| 4 (2006b) | 212 | 1087 | 13.3 | 313 | 1 |  |
|  |  |  |  |  |  |  |
|  |  |  |  |  |  |  |
| 5 (2004) | 266 | 940 | 11.3 | 155 | 0 |  |
| 5 (2005a) | 264 | 1077 | 11.1 | 173 | 0 |  |
| 5 (2005b) | 263 | 1121 | 11.6 | 211 | 0 |  |
|  |  |  |  |  |  |  |
| 6 (2004) | 289 | 940 |  | 152 | 0 |  |
| 6 (2005a) | 272 | 909 | 14.3 | 146 | 0 |  |
| 6 (2005b) |  |  | 15.5 | 158 | 0 |  |
|  |  |  |  |  |  |  |
| 7 (2002) |  |  | 13.8 | 272 | 0 |  |
| 7 (2003) |  |  | 14.4 | 277 | 0 | 4/2003-06/2005 |
| 7 (2004) |  |  | 14.2 | 259 | 1 |  |
| 7(2005) |  | 2133 | 15.5 | 315 | 1 |  |
|  |  |  |  |  |  |  |
| 8 (2003) |  |  |  |  | 0 |  |
| 8 (2004) |  |  | 11.8 | 417 | 0 |  |
| 8 (2007) |  | 1496 | 12.6 | 270 | 0 |  |
|  |  |  |  |  |  |  |
|  |  |  |  |  |  |  |
| 9 (2004) | 451 | 897 | 13.3 | 167 | 0 | 01/2004-11/2006 |
| 9 (2005) | 321 | 868 | 14 | 178 | 1 |  |
| 9 (2006a) | 282 | 1024 | 14.7 | 226 | 1 |  |
| 9 (2006b) |  |  |  | 198 | 1 |  |
|  |  |  |  |  |  |  |
| 10 (2002) | 186 | 960 |  |  | 0 |  |
| 10 (2003a) | 267 | 1129 | 13.6 | 275 | 0 | 09/2003-11/2006 |
| 10 (2003b) | 222 | 1355 | 12.4 | 216 | 1 |  |
| 10 (2004) | 259 | 1025 | 13.8 | 248 | 1 |  |
| 10 (2005) | 252 | 1246 | 13.9 | 281 | 1 |  |
|  |  |  |  |  |  |  |
| 11 (2002) | 410 | 1408 |  |  | 0 |  |
| 11 (2003) | 383 | 1478 | 14.7 | 188 | 0 |  |
| 11 (2004) | 445 | 1760 | 14.7 | 203 | 0 |  |
| 11 (2005) | 338 | 1462 | 14.6 | 214 | 0 |  |
|  |  |  |  |  |  |  |
| 12 (2003) |  |  | 14 | 286 | 0 | 05/2003-11/2004 |
| 12 (2004) |  |  | 13.8 | 273 | 1 |  |
| 12 (2005) | 490 | 1333 | 14.3 | 307 | 1 |  |
|  |  |  |  |  |  |  |
| 13 (2004) |  |  |  | 242 | 0 | 03/2004-03/2006 |
| 13 (2005) |  |  |  |  | 1 |  |
| 13 (2006) |  | 1054 | 12.1 | 329 | 1 |  |
|  |  |  |  |  |  |  |
| 14 (2005) |  |  | 15.4 | 288 | 0 |  |
| 14 (2006) |  |  |  |  | 0 |  |
| 14 (2007) |  |  | 15.7 | 318 | 0 |  |
|  |  |  |  |  |  |  |
| 15 (2002) |  |  | 14.6 | 320 | 0 |  |
| 15 (2003b) |  |  | 14.5 | 384 | 1 | 11/2002-8/2006 |
| 15 (2005) |  |  | 15.5 | 305 | 1 |  |
| 15 (2006) |  | 1146 | 14.3 | 313 | 1 |  |

When there is no entry, no data are available for that time point.
