# Supplementary material for: The Saccadic and Neurological Deficits in Type 3 Gaucher Disease
Source: PLoS One. 2011 Jul 20;6(7):e22410. doi: 10.1371/journal.pone.0022410 (PMC3140522; doi:10.1371/journal.pone.0022410)
Supplement: Table S3 — Neuropsychological testing. (DOCX) [file pone.0022410.s003.docx]

Table S3. Neuropsychological testing

|  | **Verbal, Performance, Full Scale IQ** | | | **P U R D U E P E G B O A R D *Oculo-manual dexterity scores (Dominant, non-dominant, both hands, assembly)*** | | | |
| --- | --- | --- | --- | --- | --- | --- | --- |
| **Patient (year)** | **VIQ** | **PIQ** | **FSIQ** | **z-dom** | **z-nondom** | **z-both** | **z-assemb** |
|  |  |  |  |  |  |  |  |
| 1 (2004) | 46 | 46 | 40 |  |  |  |  |
| 1 (2005) | 52 | 62 | 53 |  |  |  |  |
| 1 (2006a) |  |  | 50 | -2.68 | -3.99 | -3.22 | -2.34 |
|  |  |  |  |  |  |  |  |
| 2 (2006) | 46 | 46 | 40 | -8.73 | -7.02 | -8.19 | -4.49 |
|  |  |  |  |  |  |  |  |
| 3(2002) | 137 | 104 | 123 | -1.86 | -2.59 | -2.6 | -1.98 |
| 3(2003) |  |  |  |  |  |  |  |
| 3(2004) | 140 | 102 | 124 | -1.71 | -1.97 | 1.59 | -2.53 |
| 3 (2006) | 144 | 122 | 139 |  |  |  |  |
|  |  |  |  |  |  |  |  |
| 4 (2005) |  | 67 |  | -4.58 | -3.42 | -1.01 | -2.02 |
| 4 (2006a) |  | 64 |  |  |  |  |  |
|  |  |  |  |  |  |  |  |
| 5 (2004) | 70 | 58 | 61 |  |  |  |  |
| 5 (2005a) | 70 | 62 | 63 |  |  |  |  |
| 5 (2005b) | 80 | 71 | 73 | -3.27 | -3.05 | -1.84 | -1.56 |
|  |  |  |  |  |  |  |  |
| 6(2004) |  | 49 |  | -3.33 | -2.79 | -3.72 | -3.88 |
| 6(2005a) |  | 53 |  | -2.68 | -2.83 | -2.14 | -4.29 |
| 6(2005b) |  | 55 |  | -3.26 | -3.2 | -2.89 | -2.82 |
|  |  |  |  |  |  |  |  |
| 7(2003) | 52 | 46 | 45 | -3.72 | -2.95 | -3.07 | -2.75 |
| 7(2004) | 62 | 55 | 55 | -4.54 | -3.44 | -3.61 | -2.73 |
| 7 (2005) | 60 | 69 | 62 | -5.71 | -5.91 | -3.76 | -2.45 |
|  |  |  |  |  |  |  |  |
| 8 (2004) | 85 | 89 | 86 | -2.26 | -1.42 | -1.59 | -0.75 |
|  |  |  |  |  |  |  |  |
| 9 (2004) | 74 | 52 | 60 | -3.28 | -2.31 | 0.22 | -2.27 |
| 9 (2005) | 83 | 55 | 68 | -2.68 | -2.95 | -1.17 | -1.15 |
|  |  |  |  |  |  |  |  |
| 10 (2002) | 93 | 82 | 87 |  |  |  |  |
| 10 (2003a) | 91 | 74 | 81 |  |  |  |  |
| 10 (2003b) |  |  |  | 0.3 | -0.6 |  | -0.22 |
| 10 (2004) | 88 | 73 | 78 | -1.51 | -1.92 | -2.8 | -0.87 |
| 10 (2005) | 88 | 74 | 79 | -2.5 | -4.46 | -3.5 | -1.69 |
|  |  |  |  |  |  |  |  |
| 11 (2002) | 101 | 102 | 101 |  |  |  |  |
| 11 (2003) | 105 | 102 | 104 |  |  |  |  |
| 11 (2004) | 111 | 111 | 112 | -1.39 | -1.44 | -2.04 | -6.47 |
| 11 (2005) | 101 | 104 | 102 |  |  |  |  |
|  |  |  |  |  |  |  |  |
| 12 (2003) | 117 | 92 | 107 | -1.71 | -2.61 | -2.67 | -0.56 |
| 12 (2004) | 112 | 102 | 108 | -2.63 | -4.52 | -1.96 | -2.13 |
| 12 (2005) | 111 | 109 | 110 | -1.57 | -1.61 | -3.73 | -2.11 |
|  |  |  |  |  |  |  |  |
| 13 (2004) | 69 | 75 | 69 |  |  |  |  |
| 13 (2005) | 71 | 69 | 67 |  |  |  |  |
| 13 (2006) | 69 | 86 | 75 | -0.49 | 0.24 | 0.96 | -0.03 |
|  |  |  |  |  |  |  |  |
| 14 (2005) | 75 | 62 | 66 |  |  |  |  |
| 14 (2007) | 70 | 64 | 65 |  |  |  |  |
|  |  |  |  |  |  |  |  |
| 15 (2002) | 89 | 68 | 77 | -4.3 | -4.3 | -4.2 |  |
| 15 (2003) | 92 | 69 | 82 |  |  |  |  |
| 15 (2005) | 95 | 76 | 86 | -2.6 | -2.06 | -5.06 | -2.56 |
| 15 (2006) | 93 | 80 | 87 |  |  |  |  |
