# Supplementary material for: The Saccadic and Neurological Deficits in Type 3 Gaucher Disease
Source: PLoS One. 2011 Jul 20;6(7):e22410. doi: 10.1371/journal.pone.0022410 (PMC3140522; doi:10.1371/journal.pone.0022410)
Supplement: Table S4 — Neurophysiological characteristics. Legend for BAER score: 0-Normal absolute & normal interpeak interval 1-1- Late absolute, normal interpeak interval 2- Late absolute, Prolonged interpeak interval 3- No peaks after wave 3 4- No peaks after waves 1 or 2 5- No peaks Legend for EEG score: 0- normal 1- Diffuse background slowing without low voltage 2- Diffuse irregular background slowing with low voltage 3- Sharps and/or spike/slow wave complex(es) (diffuse and or lateralized) 4- 1+3 5- 2+3 PCS: partial complex seizures. (DOCX) [file pone.0022410.s004.docx]

**Table S4. Neurophysiological characteristics**

| **Patient** | **Seizure type** | **Year** | **BAER Score (L)** | **BAER Score (R)** | **SSEP (amplitude P22-N35)- µV** | **Hammer Amplitude (µV)** | **EEG score** |
| --- | --- | --- | --- | --- | --- | --- | --- |
|  |  |  |  |  |  |  |  |
| 1 | Complex partial seizure | 2004 | 4 | 2 | 0.6 | 4.95 | 1 |
|  |  | 2005 | 5 | 4 | 1.1 | 4.95 | 5 |
|  |  | 2006a | 5 | 5 | 4.6 | 7.6 | 5 |
|  |  |  |  |  |  |  |  |
| 2 | Myoclonic | 2004 | 4 | 4 | 33.8 | 30.35 | 5 |
|  |  | 2005 | 4 | 4 | 28.3 | 26.3 | 5 |
|  |  | 2006 | 4 | 4 | 53.6 | 11.7 | 5 |
|  |  |  |  |  |  |  |  |
| 3 | None | 2002 | 4 | 4 | n.d. | n.d. |  |
|  |  | 2003 | 4 | 3 | 3.6 | 4.6 | n.d. |
|  |  | 2004 | 3 | 4 | 3.8 | 4.8 | 0 |
|  |  | 2006 | 4 | 4 | 3.6 | 3.6 | 0 |
|  |  |  |  |  |  |  |  |
| 4 | None | 2004 | 0 | 0 | 50.7 | 58 | 3 |
|  |  | 2005 | 0 | 0 | 51 | 44.5 | 3 |
|  |  | 2006a | 0 | 0 | 44 | 47 | 5 |
|  |  | 2006b | 0 | 0 | 16 | 9.65 | n.d. |
|  |  |  |  |  |  |  |  |
| 5 | None | 2004 | 0 | 4 | 7.5 | 4.4 | 1 |
|  |  | 2005a | 3 | 3 | 5.7 | 7.7 | n.d. |
|  |  | 2005b | 2 | 2 | 8 | 4.8 | 1 |
|  |  |  |  |  |  |  |  |
| 6 | Generalized tonic clonic | 2004 | 3 | 3 | 4.2 | 7.1 | 4 |
|  |  | 2005a | 4 | 3 | 1.6 | 3.24 | 4 |
|  |  | 2005b (span only) | n.d. | n.d. | 3.3 | 8.95 | 4 |
|  |  |  |  |  |  |  |  |
| 7 | None | 2002 | 5 | 5 | n.d. | 1.6 | 4 |
|  |  | 2003 | 3 | 3 | n.d. | 6.2 | 3 |
|  |  | 2004 | 3 | 4 | 13.7 | 2.7 | 4 |
|  |  | 2005 | 3 | 4 | 6.3 | 3.35 | 1 |
|  |  |  |  |  |  |  |  |
| 8 | None | 2003 | n.d. |  | n.d. | n.d. | 2 |
|  |  | 2004 | 0 | 0 | 1.51 | n.d. | n.d. |
|  |  |  |  |  |  |  |  |
| 9 | None | 2004 | 5 | 4 | 1.7 | 1.8 | 4 |
|  |  | 2005 | 4 | 4 | 4.1 | 1.95 | 5 |
|  |  | 2006a | 4 | 5 | 1.7 | 4 | 1 |
|  |  | 2006b | 4 | 4 | 1.3 | 1.55 | 2 |
|  |  |  |  |  |  |  |  |
| 10 | None | 2002 | 0 | 0 | n.d. | 2.9 | 0 |
|  |  | 2003a | 0 | 0 | 6 | 11.5 | n.d. |
|  |  | 2003b | 0 | 0 | 6.8 | 7 | n.d. |
|  |  | 2004 | 0 | 0 | 9.2 | 8.5 | 1 |
| 10 | None | 2005 | 0 | 0 | 8.2 | 6.8 | 0 |
|  |  | 2006a | 0 | 0 | 6.7 | 5.95 | n.d. |
|  |  | 2006b | 0 | 0 | n.d. | 2.8 | 0 |
|  |  |  |  |  |  |  |  |
| 11 | None | 2002 |  |  | 2.7 | 2.5 | n.d. |
|  |  | 2003 | 0 | 0 | 3.3 | 3.5 | 2 |
|  |  | 2004 | 0 | 0 | 1.7 | 2.7 | 0 |
|  |  | 2005 | 0 | 0 | 1.75 | 5.1 | 0 |
|  |  |  |  |  |  |  |  |
| 12 | None | 2003 | 0 | 0 | 3.8 | 3.3 | 1 |
|  |  | 2004 | 4 | 3 | 3 | 3.5 | 2 |
|  |  | 2005 | 4 | 4 | 4.6 | 4.3 | 2 |
|  |  |  |  |  |  |  |  |
| 13 | Generalized tonic clonic | 2006 | 3 | 3 | 3.8 | 4.1 | 4 |
|  |  |  |  |  |  |  |  |
| 14 | None | 2005 | 4 | 4 | 6.2 | 7.1 | 1 |
|  |  | 2007 | n.d. | n.d. | n.d. | n.d. | 1 |
|  |  |  |  |  |  |  |  |
| 15 | None | 2002 | 2 | 1 | n.d. | 1.79 | n.d. |
|  |  | 2003b | 0 | 5 | 2.8 | 1.4 | n.d. |
|  |  | 2005 | 0 | 5 | 2.5 | n.d. | 2 |
|  |  | 2006 | 2 | 0 | 1.6 | 0.6 | 2 |

Legend for BAER score:

1. Normal absolute & normal interpeak interval
2. 1- Late absolute, normal interpeak interval

2- Late absolute, Prolonged interpeak interval

3- No peaks after wave 3

4- No peaks after waves 1 or 2

5- No peaks

Legend for EEG score:

0- normal

1- Diffuse background slowing without low voltage

2- Diffuse irregular background slowing with low voltage

3- Sharps and/or spike/slow wave complex(es) (diffuse and or lateralized)

4- 1 + 3

5- 2 + 3

PCS: partial complex seizures
