# Supplementary material for: The Saccadic and Neurological Deficits in Type 3 Gaucher Disease
Source: PLoS One. 2011 Jul 20;6(7):e22410. doi: 10.1371/journal.pone.0022410 (PMC3140522; doi:10.1371/journal.pone.0022410)
Supplement: Protocol S1 — Protocol Title: Clinical and Biochemical Effects of Macrophage-Targeted Glucocerebrosidase on Neurological Involvement in Neuronopathic Gaucher Disease'. (DOC) [file pone.0022410.s006.doc]

Date: June 30, 2007

Protocol Number: 91-N-0225

Protocol Title: **Clinical and Biochemical Effects of Macrophage-Targeted Glucocerebrosidase on Neurological Involvement in Neuronopathic Gaucher Disease’**

Principal Investigator: Raphael Schiffmann, DMNB, Bldg. 10, Rm. 3D03, NINDS, 301-496-1465, [RS4e@NIH.GOV](mailto:RS4e@NIH.GOV)

Associate Investigators:

# Margaret Timmons DMNB, Bldg. 10, Rm. 3D03, NINDS, 301-496-1465 **mt308f@nih.gov**

William Benko Askari DMNB, Bldg. 10, Rm. 3D03, NINDS, 301-496-1465 benkow@ninds.nih.gov

Gary Murray, PhD. , Bldg. 10, Rm. 3D03, NINDS, 301-496-1465, [murrayg@ninds.nih.gov](mailto:murrayg@ninds.nih.gov)

, M.D., Section on Molecular Neurogenetics, National Institute of Mental Health and National Human Genome Research Institute, Building 35, Room 1A213, 35 Convent Drive, MSC 3708, Bethesda, Maryland 20892-3708 email: sidranse@irp.nimh.nih.gov

Research Contact: Chevalia (Val) Robinson DMNB, Bldg. 10, Rm. 3D03, NINDS,301-496-1465 cr163w@nih.gov

Collaborating Sites: None

Number and Type of Subjects: 70 patients with neuronopathic Gaucher disease

Estimated duration of study: 3 years

Product Uses Ionizing Radiation: YES medical use only

Project Uses IND/IDE: No

Project Uses “Durable Power of Attorney”: No

On/Off Site: No

Multi-Institutional Project: No

**CLINICAL RESEARCH PROJECT: Title of Project: Clinical and Biochemical Effects of Macrophage-Targeted Glucocerebrosidase on Neurological Involvement in Neuronopathic Gaucher's Disease. 91-N-0225**

**1.0 PRECIS**

The purpose of this study is to examine the effects of enzyme replacement therapy in patients with neuronopathic Gaucher's disease and to investigate the pathogenesis of their neurological signs and symptoms. Macrophage-targeted glucocerebrosidase will be administered by intravenous infusion under the supervision of the patient's private physician at an initial dosage of 60 to 120 IU per kg of body weight weekly or every other week. Patients will be categorized as treatment responders if they display a clinically significant increase in hemoglobin concentration and a reduction in hepatic or splenic volume. Improvement in these parameters over time will be correlated with measurements for metabolic encephalopathy and radiologic, electrophysiologic and psychometric measurements of neurological involvement.

**2.0 INTRODUCTION AND SCIENTIFIC BACKGROUND**

Gaucher's disease is the most prevalent of the lysosomal storage disorders. It is caused by deficiency of glucocerebrosidase activity with secondary accumulation of glucocerebroside within the lysosomes of macrophages. The storage process produces a multisystem disease that includes progressive visceral enlargement and gradual replacement of the bone marrow with distinctive lipid-laden macrophages. Symptomatic anemia, coagulation abnormalities, hepatosplenomegaly and structural skeletal alterations occur in most patients with the chronic visceral form of the illness^1^. The latter is referred to as type 1 Gaucher's disease and is the most frequently encountered phenotype.

Progressive neurological deterioration occurs in a minority of patients^2^.Type 2 Gaucher's disease is an acute neuronopathic form of the illness that presents during infancy between three and six months of age. In addition to typical systemic signs of Gaucher's disease, a characteristic neurological syndrome develops that includes persistent hyperextension of the head, strabismus, trismus, generalized spasticity and psychomotor regression. Death usually occurs by two years of age as a result of progressive brainstem dysfunction.

Type 3 Gaucher's disease is a chronically progressive neuronopathic form of the illness that presents during infancy, childhood, adolescence or adulthood. The clinical features of this phenotype are more variable than those observed in Type 2 Gaucher's disease. At least three subtypes are distinguishable on clinical grounds. The most thoroughly characterized group of patients with type 3 Gaucher's disease presents during childhood, adolescence or early adulthood with progressive myoclonic epilepsy in conjunction with a horizontal supranuclear gaze palsy. Dementia, ataxia and spasticity develop as the illness progresses over several years. Systemic signs of Gaucher's disease tend to be mild with death occurring as a result of progressive neurological deterioration. Neuropathological examination provides clear evidence of nerve cell loss and neuronophagia particularly within the brainstem^3^.

The Norrbottnian isolate in Northern Sweden represents another carefully studied group of patients with type 3 Gaucher's disease. Presentation typically occurs in childhood with aggressive systemic disease and a slowly progressive neurological syndrome consisting of horizontal supranuclear gaze palsy, dementia, ataxia, spasticity and occasional generalized seizures without myoclonus^4^. Death usually occurs in adolescence as a result of systemic complications of the disease. Bone marrow transplantation has been reported to arrest the systemic illness^5^ and possibly prevent progression of cognitive impairment in these patients^6^.

Over the past decade, we have identified a third group of patients with type 3 Gaucher's disease^7^. In our experience, this is the most prevalent, albeit rare, neuronopathic phenotype with less than 1 case per 100,000 people. The distinctive features of the illness include: a) presentation in infancy or childhood with very aggressive systemic disease leading to death in adolescence from complications of portal or pulmonary hypertension, b) early development of horizontal supranuclear gaze palsy as the major neurological sign of the illness, and c)infrequent seizures usually as agonal events. Detailed neurological evaluation of our case material revealed a variable combination of electroencephalographic abnormalities, abnormal brainstem auditory evoked potentials and mild cognitive impairment. The latter included difficulties with recall of abstract, visually present symbols and acquisition of math and spelling skills. Prior to the development of enzyme replacement therapy, clear progression of the neurological syndrome could not be demonstrated before patients succumbed to systemic disease. After three years of treatment in a small cohort of patients, this issue remains unresolved.

The neuropathological features of this phenotype are poorly defined. Autopsy data are limited to a single case report in which the only morphological abnormality was infiltration of the Virchow-Robin spaces with lipid-laden macrophages^8^. The latter cells are derived from vascular adventitial histiocytes and are present in both the neuronopathic and non-neuronopathic forms of Gaucher's disease^3^. Neuronophagia and microglial nodules were not observed despite an exhaustive examination. Since a direct neuronal insult could not be demonstrated, toxic or metabolic factors extrinsic to the neuron must be considered in further studies of the pathogenesis of this syndrome.

The purpose of the present investigation is to determine the effects of enzyme replacement therapy in patients with neuronopathic Gaucher disease and to examine the pathogenesis of the associated neurological signs and symptoms. The following issues will be addressed: a) Does severe systemic disease produce a "metabolic encephalopathy" in these patients? b) Does a cytotoxic metabolite of glucocerebroside known as glucopsychosine (glucosylsphingosine)^9,10^ accumulate within the vascular and spinal fluid compartments? Is the level of this metabolite correlated with the severity of the neurological syndrome? c) Since Gaucher cells actively secrete cytokines^11,12^, could measurement of the latter, in particular interleukin 1, within the spinal fluid provide a measure of the extent of neurological involvement? d) Does reversal of the systemic illness with enzyme replacement therapy^13-15^ correlate with changes in any of these potential "markers" of neurological involvement? e) Does cognitive dysfunction stabilize after systemic disease is reversed with enzyme replacement therapy?

**3.0 STUDY DESIGN**

**3.1 PATIENT SELECTION CRITERIA**

1. All patients with neuronopathic Gaucher's disease who have a partial or complete horizontal supranuclear gaze palsy or a genotype associated with neurological involvement will be considered as potential candidates for this study.

2. All candidates must be serologically nonreactive for hepatitis B and human immunodeficiency (AIDS) virus. HIV positive patients will be excluded because of the effects of the latter illness on cognitive performance.

3. Individuals with neoplastic disease will be excluded.

4. The general health and well being of each candidate must be sufficient to allow for a modest amount of blood drawing, collection of appropriate urine and spinal fluid specimens and performance of necessary roentgenographic and magnetic resonance (MR) imaging studies. In addition, each candidate must be able to return to the National Institutes of Health (NIH) on a regular basis dictated by disease severity for monitoring of laboratory parameters.

**3.2 EXCLUSION CRITERIA**

1. Patient who participates in a clinical study of an investigational therapeutic agent for Gaucher Disease.

2. Patient and /or the patient’s parent(s) or legal guardian(s) are unable to understand the nature, scope, and possible consequences of the study.

3. Patient is unable to comply with the protocol, e.g., uncooperative with protocol schedule, refusal to agree to all of the study procedures.

**3.3 CLINICAL AND LABORATORY EVALUATIONS**

Potential candidates for this study will be admitted to the Clinical Center for a maximum of ten days. During this time, they will be evaluated by a carefully recorded history and physical examination and screening laboratory studies as outlined in the appended patient testing schedule. The latter will include a complete blood count, routine serum chemistries, hepatitis B and HIV serology, serum iron panel, coagulation profile, routine urinalysis, chest x-ray and electrocardiogram. A skin biopsy will be performed in order to establish fibroblast cultures for confirmation of diagnosis by enzyme assays and genotype analyses. Other clinical appraisals will be performed as indicated in order to evaluate the medical condition of the patient. These tests will be used for preliminary assessment of the patient's health and whether it is medically advisable that they participate in the study.

**Patient Testing Schedule (6-12 Month Intervals)**

1. EKG 2. Chest and Spine X-rays

3. Chest CT Scan 4. MRI-Head

5. MRI-Liver and Spleen 6. Abdominal Ultrasound

7. MRI-Spine/Hips/Knees/Ankles 8. Eye Movement Recording

9. Psychometric Evaluation 10. Electroencephalogram

11. Auditory Evoked Potentials

12. HIV 13. Hepatitis B_s_ Antigen

14. Cerezyme Antibody 15. Thyroid Panel

16. PTH (Intact) 17. ACTH (Fasting)

18. Cortisol (Fasting) 19. Serum Iron

20. Transferrin/% Saturation 21. Ferritin

22. Serum folate 23. Vitamin B_12_

24. Routine Urinalysis

25. Vitamins A, C and E 26. Venous Blood Gas

27. Serum Ammonia 28. Serum Mg and Zn

29. CBC/Platelets 30. Retic Count/Sed Rate

31. PT/PTT 32. Acute Care Panel

33. ACE/Acid Phosphatase 34. Hepatic Panel

35. Plasma Glucocerebroside 36. Plasma Glucopsychosine

Overall disease severity will be evaluated both radiologically and physiologically. The extent of skeletal involvement and the degree of infiltration of the bone marrow by storage material will be evaluated on radiograms of the spine and long bones and T_1_-weighted MR scans of the lumbar spine and lower extremities, respectively. The volume of the spleen and liver will be quantitated on abdominal MR images. The extent of interstitial lung disease will be qualitatively assessed on chest CT scans. The presence of portal hypertension will be determined by ultrasound examination of portal vein size and direction of blood flow.

Laboratory evaluations for metabolic encephalopathy will include measurement of the venous blood gas, serum ammonia concentration and thyroid, parathyroid and adrenal function in addition to routine serum chemistries. Total amount of blood is estimated to be 35 ml. No more than 3 ml/kg of blood will be drawn in a single visit.

Neurological evaluation will include a cranial MR scan, an electroencephalogram, brainstem auditory evoked potentials, a detailed neuro-ophthalmological examination with eye movement recordings and a psychometric evaluation with standardized test instruments (Wechsler Pre-School and Childrens' Scales, McCarthy Scales, Woodcock-Johnson Psychoeducational Battery and Peabody Individual Achievement Tests). In addition to routine studies, spinal fluid specimens will be analyzed for interleukin 1, glucocerebroside^17^ and glucopsychosine^18^ content and glucocerebrosidase and chitotriosidase^19^ activity.

Patients who fulfill the selection criteria and elect to participate in the study will undergo periodic inpatient re-evaluations at the Clinical Center. The frequency of re-evaluation will be every six to twelve months and will be based on overall disease severity. The clinical and laboratory examinations described above will be obtained during each admission.

**3.4 ENZYME INFUSIONS**

All patients with type 3 Gaucher's disease who are currently receiving infusions of macrophage-targeted glucocerebrosidase on an outpatient basis at NIH will be transferred to the care of their private physicians in 1995. The private physician will then assume responsibility for management of each patient's treatment. Enzyme dosage recommendations will be based on the results of the inpatient evaluation at NIH and will generally lie in the range of 60-120 units per kilogram of body weight weekly or every other week as a function of disease severity and the response to treatment. Initiation and management of enzyme replacement therapy for all patients newly enrolled in this study will be the responsibility of the referring physician. Clinical decisions regarding enzyme infusions will be made by the primary physician in consultation with the principal investigator of this protocol.

**3.5 RESPONSE CRITERIA**

Patients will be categorized as responders to enzyme replacement if they display a clinically significant increase in

hemoglobin concentration and a reduction in hepatic or splenic volume. A significant increase in hemoglobin concentration is defined as a value that is consistently 1 g/dl greater than pretreatment values. With regard to hepatic and splenic volume, a reduction in excess of 12 % of the pretreatment value is significant. Improvement in these parameters over time will be correlated with measurements of cardiopulmonary function, laboratory examinations for metabolic encephalopathy and radiologic, electrophysiologic and psychometric measurements of neurological involvement. The potential for clinically significant change in any of the latter parameters over the proposed period of study is unknown at the present time.

**4.0 DRUG AVAILABILITY**

CEREDASE^R^ glucocerebrosidase is commercially available from Genzyme Corporation, Cambridge, MA. The purified enzyme is supplied as a sterile, non-pyrogenic solution of mannose-terminated, human placental glucocerebrosidase in 1 % USP human serum albumin. Recently, CEREZYME^R^ glucocerebrosidase, a mannose-terminated form of the enzyme prepared by recombinant DNA technology, has also become commercially available from Genzyme Corporation. The latter material is supplied as a lyophilized powder that is reconstituted with USP normal saline prior to infusion. Although quantities of the recombinant material are limited at present, increased manufacturing capability is anticipated in 1995. Eventually, the

placental product will be replaced completely by the recombinant material.

**5.0 DATA COLLECTION**

The specific patient information and laboratory data to be collected during the study will be recorded on standardized case record forms and entered into a computerized data base. Dr. James Dambrosia will be responsible for overall management of the data base and for statistical analysis of the data generated during the study.

**6.0 PROTECTION OF HUMAN SUBJECTS**

**6.1 Rationale for Subject Selection**

The selection of subjects with neuronopathic Gaucher disease is part the main goal of the study.

**6.2** **Justification for Exclusions**

No patient is excluded on the basis or sex or age. The other exclusion criteria are self explanatory.

**6.3** **Participation of vulnerable subjects**

Children are included since this is the largest age group with neuronopathic Gaucher disease. Gaucher disease may cause physical or cognitive impairment.

**6.4 RISKS, DISCOMFORTS AND INCONVENIENCES**

1. Patients who enroll in this study will undergo initial evaluation and periodic monitoring of clinical and laboratory parameters on an inpatient basis at the Clinical Center. Hospitalization for a maximum of ten days will occur at six to twelve month intervals. Each admission will focus on the extent and severity of the visceral and neurological disease and whether it has responded to treatment. Psychometric tests will be performed on either an inpatient or outpatient basis as dictated by patient convenience.

2. Measurement of the clinical and laboratory parameters necessary for this study is associated with limited risks and discomforts. During inpatient evaluations ≤ 35 ml of blood will be withdrawn by venipuncture. Venipuncture causes some pain of brief duration at the phlebotomy site. There is also a remote chance of infection and bleeding into the tissues. Radiographic visualization of the chest, spine and long bones results in exposure to x-rays. These examinations are medically indicated and will be performed every six to twelve months. Some patients may experience transient claustrophobia during MR scanning procedures. Psychometric evaluation may be associated with a sense of stress and anxiety during the testing procedures. Electroencephalographic and evoked potential studies may be associated with slight discomfort that arises from the odor of the paste required to secure the electrodes to the scalp and the cleaning solution used for their removal.

4. Cerezyme is the standard care for type 1 and 3 Gaucher disease. Current data indicate that approximately 15-20% of patients treated with CEREZYME^R^ glucocerebrosidase develop antibodies to the enzyme^20^,^21^. All antibodies detected to date have been of the I_g_G class and developed within the first year of treatment. In general, treatment responses are not attenuated by the presence of antibody with a single known exception. One of the children enrolled in the present study developed a neutralizing antibody associated with rapid progression of underlying disease. Immune tolerance was induced in this patient by a course of intravenous immunoglobulin, cytotoxic drug administration and high dose infusions of antigen (CEREZYME^R^. If additional patients develop neutralizing antibodies, they may withdraw from the study at any time and a protocol for induction of immune tolerance will be provided to the primary physician. Approximately 2% of seropositive patients experience periodic symptoms suggestive of immediate hypersensitivity. These symptoms are readily controlled by premedication with antihistaminic agents. None of the patients treated to date have developed I_g_E antibodies. In order to monitor for the development of antibodies to CEREZYME^R^ glucocerebrosidase during the present study, serum specimens will be analyzed by ELISA assay prior to and every six months after enzyme infusions have begun.

5. Skin biopsy for fibroblasts culture will be performed once only. In 5% of the cases the cells do not grow in the laboratory and a second biopsy is required.

6. Saccadic Eye Movement Assessments: Saccadic eye movement assessments will be conducted during each visit. All patients with neuronopathic Gaucher disease have a supranuclear gaze palsy consisting of slow horizontal saccades, and to a lesser extent slow vertical saccades. Both vertical and horizontal saccades shall be measured where possible. The number of trials required for each measurement and the equipment used shall be dependent on each patient’s age, ability to co-operate and follow instructions and shall be at the assessor’s discretion. This is a non-invasive test with minimal discomfort.

**6.5 Evaluation of Benefits and Risks/Discomforts**

1. Benefits: The research involves the prospect of direct benefit to individual subjects. Patients will benefit from care by a group that is expert in this disorder that is likely to make specific recommendations regarding optimal therapy of this rare disease.

2. Risks: The research involves no more than minimal risk to subjects. All tests are standard care that patients often undergo routinely. .

3. Risk/benefit ratio: In view of the fact that the tests that are performed are all standard care for this patient population, the risks are reasonable in relation to the anticipated benefits.

**6.6 Alternatives to Participation or Alternative Treatments**

Alternative to participation is not participating. Treatment with Cerezyme being stadard medical care, the patient will continue this therapy even if he/she is not participating in this study. (List approved therapies or treatments for clinical trials.

**6.7 Protocol Consent/Assent Processes and Documents**

Each subject will receive an oral and written explanation of the purposes, procedures, and risks of this study in language appropriate for the individual’s level of understanding. A copy of the signed consent form will be placed in the medical record. A member of the protocol team will be available to answer questions about the study to be performed. Consent will be obtained by the Principal or Associate Investigator.

**6.8 Patient Advocate**

A patient’s rights representative, is available to patients on this protocol. The representative is located in Building 10 and can be reached by phone at 301-496-2626. Patients may ask any questions about the study and may withdraw their consent at any time.

**6.9 Confidentiality**

All medical information collected from study participants will be kept in a locked file at the NIH Clinical Center. Unique patient identifiers will be used to label all data. Strict standards of confidentiality will be upheld at all times.

### 6.9.1 Conflict of Interest/ Technology Transfer

**a.** Statement of any conflict of interest of any investigator: no investigator on this protocol has any conflict of interest of any kind.

**b.** List tech transfer agreement/s: none

**c.** Statement of what drug company/ sponsor will provide: N/A

**7. Adverse Event Reporting**

All adverse events will be reported by the PI to the IRB within 7 days.

Stopping criteria: This study will be temporarily stopped/terminated if there are two (2) SAEs that are life-threatening or fatal which are judged to be possibly/probably related to this research.

Subject withdrawal: If a patient or the patient’s parent(s) or legal guardian(s) withdraw consent or the patient is withdrawn from the study prematurely, the investigator will complete the appropriate report describing the reason for discontinuation. Patients withdrawing or discontinuing prematurely from the study for any reason will not be replaced. A patient may be withdrawn from the study for the following medical or administrative reasons:

**Adverse Event**: If a patient suffers an AE, which, in the judgment of the investigators presents an unacceptable consequence or risk to the patient, the patient may be withdrawn from further participation in the study.

**Adverse Laboratory Event**: If a patient has an adverse laboratory event, which, in the judgment of the investigator, the study sponsor, or the medical monitor, presents an unacceptable consequence or risk to the patient, the patient may be withdrawn from the study.

**Intercurrent Illness**: If a patient develops an illness during the course of the study that is not associated with the condition under study and which requires treatment that is not consistent with protocol requirements, the patient may be withdrawn from the study. A patient may also be withdrawn from the study if, in the judgment of the investigators, he develops an intercurrent illness that in any way justifies his/her withdrawal.

**Administrative Withdrawal**: After consultation, a patient may be withdrawn from the study for the following administrative reasons: (1) failure to visit the clinic at the scheduled dates or (2) failure to comply with protocol requirements.

**8.0 Data and Safety Monitoring Plan**

In view of the low risks to subjects participating in this protocol, continuous, close monitoring by the Principal Investigator shall constitute the data and safety monitoring plan for this protocol.

**9.0 BIBLIOGRAPHY**

1. Brady RO and Barranger JA. Glucosylceramide lipidosis: Gaucher's disease. In: Stanbury JB, Wyngaarden JB, Fredrickson DS, Goldstein JL and Brown MS, eds. The metabolic basis of inherited disease. 5th ed. McGraw-Hill, New York, 1983, pp 842-56.

2. Patel SC, Barton NW and Argoff C. Niemann-Pick disease, types A, C and D, Gaucher's disease, types 2 and 3 and Wolman's disease. In: deJong JMBV, ed. Vol 16 (60) of Handbook of clinical neurology. Elsevier Science Publishers, Amsterdam, 1991, pp 147-64.

3. Winkelman MD, Banker BQ, Victor M and Moser HW. Non-infantile neuronopathic Gaucher's disease: a clinicopathologic study. Neurology 1983; 33: 994-1008.

4. Erikson A. Gaucher disease-Norrbottnian type (III): neuropaediatric and neurobiological aspects of clinical patterns and treatment. Acta Paediatr Scand Suppl 1986; 326: 1-42.

5. Ringdén O, Groth CG, Erikson A et al. Long-term follow-up of the first successful bone marrow transplantation in Gaucher disease. Transplantation 1988; 46: 66-70.

6. Erikson A, Groth CG, Månsson JE, Percy A, Ringdén O and Svennerholm L. Clinical and biochemical outcome of marrow transplantation for Gaucher disease of the Norrbottnian type. Acta Pediatr Scand 1990; 79: 680-5.

7. Patterson MC, Horowitz M, Abel RB et al. Isolated horizontal supranuclear gaze palsy as a marker of severe systemic involvement in Gaucher's disease. Neurology 1993; 43: 1993-7.

8. Kaye EM, Ullman MD, Wilson ER and Barranger JA. Type 2 and type 3 Gaucher disease: a morphological and biochemical study. Ann Neurol 1986; 20: 223-30.

9. Conradi NG, Sourander P, Nilsson O, Svennerholm L and Erikson A. Neuropathology of Norrbottnian type of Gaucher disease: morphological and biochemical studies. Acta Neuropathol 1984; 65: 99-109.

10. Conradi NG, Kalimo H and Sourander P. Reactions of vessel walls and brain parenchyma to the accumulation of Gaucher cells in the Norrbottnian type (type 3) of Gaucher disease. Acta Neuropathol 1988; 75: 385-90.

11. Gery I, Zigler S, Brady RO and Barranger JA. Selective effects of glucocerebroside (Gaucher's storage material) on macrophage cultures. J Clin Invest 1981; 68: 1182-89.

12. Dinarello CA and Mier JW. Lymphokines. N Engl J Med 1987; 317: 940-5.

13. Barton NW, Furbish FS, Murray GJ, Garfield M and Brady RO: Therapeutic response to intravenous infusions of glucocerebrosidase in a patient with Gaucher disease. Proc Natl Acad Sci USA 1990; 87: 1913-6.

14. Barton NW, Brady RO, Dambrosia JM et al. Replacement therapy for inherited enzyme deficiency: macrophage-targeted glucocerebrosidase for Gaucher's disease. N Engl J Med 1991; 324: 1464-70.

15. Barton NW, Brady RO, Dambrosia JM et al. Dose-dependent responses to macrophage-targeted glucocerebrosidase in a child with Gaucher's disease. J Pediatr 1992; 120: 277-80.

16. Parker RI, Grewal RP, McKeown LP and Barton NW. The effect of platelet count on the DDAVP-induced shortening of the bleeding time in thrombocytopenic Gaucher's patients. Am J Pediatr Hematol Oncol 1992; 14: 39-43.

17. Ullman MD and McCluer RH. Quantitative analysis of plasma neutral glycosphingolipids by high performance liquid chromatography of perbenzoyl derivatives. J Lipid Res 1977; 18: 371-8.

18. Shinoda H, Kobayashi T, Katayama M, Goto I and Nagara H. Accumulation of galactosylsphingosine (psychosine) in the twitcher mouse: determination by HPLC. J Neurochem 1987; 49: 92-9.

19. Hollak CEM, van Weely S, van Oers MHJ and Aerts JMFG. Marked elevation of plasma chitotriosidase activity: a novel hallmark of Gaucher disease. J Clin Invest 1994; 93: 1288-92.

20. Richards SM, Olson TA and McPherson JM. Antibody response in patients with Gaucher disease after repeated infusion of macrophage-targeted glucocerebrosidase. Blood 1993; 82: 1402-9.

21. Grabowski GA, Barton NW, Pastores G et al. Enzyme therapy in Gaucher disease type 1: comparative efficacy of mannose-terminated glucocerebrosidase from natural and recombinant sources. Ann Intern Med 1995; 122: 33-9.
